# Renal and Hepatic Toxicities After Pressurized Intraperitoneal Aerosol Chemotherapy (PIPAC)

**DOI:** 10.1245/s10434-012-2840-2

**Published:** 2013-02-03

**Authors:** Ana Blanco, Urs Giger-Pabst, Wiebke Solass, Jürgen Zieren, Marc A. Reymond

**Affiliations:** 1 Department of Surgery, Marienhospital Herne, Ruhr University Bochum, Bochum, Germany; 2 Institute of Pathology, Bergmannsheil, Ruhr-University Bochum, Bochum, Germany; 3 Chirurgische Klinik, Marienhospital Herne, Universitätsklinikum der Ruhr-Universität Bochum, Herne, Germany

## Abstract

**Background:**

Both in animal models and in human patients, pressurized intraperitoneal aerosol chemotherapy (PIPAC) has been shown to improve local bioavailability of chemotherapy in peritoneal nodules, as compared with conventional peritoneal lavage. Pharmacokinetic studies show a low drug concentration in peripheral venous blood. However, hepatic and renal toxicities induced by delivering chemotherapeutic drugs into the abdomen as a pressurized aerosol have not yet been investigated.

**Methods:**

Liver and renal function as well as toxicity parameters were monitored after eight PIPAC applications with doxorubicin (1.5 mg/m^2^ body surface) and cisplatin (7.5 mg/m^2^ body surface) in three end-stage patients suffering therapy-resistant peritoneal carcinomatosis. PIPAC was repeated at 4-week intervals (three times in two patients, twice in one patient). Peripheral venous blood was collected preoperatively and then daily until the 5th postoperative day, and sent to the hospital’s clinical chemistry laboratory. Statistical analysis was performed by analysis of variance (ANOVA).

**Results:**

Gamma-glutamyltransferase was significantly elevated (*p* < 0.05) in the early postoperative phase. Glutamic oxaloacetic transaminase [aspartate aminotransferase], glutamic pyruvic transaminase [alanine aminotransferase], and bilirubin levels were not influenced by the procedure. Quick-test remained normal. Serum creatinine levels were not altered.

**Conclusions:**

Under the above conditions, PIPAC did not induce clinically relevant liver cytotoxicity. Liver metabolism and function were not altered. Renal function remained within the normal range. No cumulative toxicity was observed after repeated PIPAC. PIPAC appears to be associated with very limited hepatic and renal toxicity, which might be a significant advantage over other administration routes.

In spite of significant progress in chemotherapy regimens, peritoneal carcinomatosis (PC) still has poor prognosis and remains an unmet medical need. Systemic chemotherapy is the standard therapy in this palliative situation, but survival benefit is limited, with for example median survival of 22 months in recurrent ovarian cancer and 16 months in colorectal cancer.^1^–^3^ The limited results of chemotherapy are explained in part by the mechanisms of chemoresistance in these advanced tumors but also by poor penetration of the therapeutic substance into tumor tissue.^4^,^5^

Over the last decade, locoregional delivery of chemotherapy into the abdominal cavity has been increasingly applied for treating PC, with the aim of increasing the drug concentration ratio between tumor cells and plasma compartment. This approach has been validated by pharmacological studies, and more recently by clinical studies, for example, in ovarian cancer and colorectal cancer.^4^,^6^–^8^

However, the efficacy of intraperitoneal chemotherapy is impaired by two main pharmacological limitations, namely poor penetration into tumor nodules and limited distribution within the abdomen.^6^ Therefore, prior complete surgical cytoreduction is required for effective intraperitoneal chemotherapy.^9^

Pressurized intraperitoneal aerosol chemotherapy (PIPAC) is an innovative technique, applying chemotherapeutic drugs as a pressurized aerosol into the abdomen during laparoscopy.^10^ In an animal model and ex vivo in surgical specimens, PIPAC has been shown to improve the local bioavailability of drug and staining substances, as compared with conventional peritoneal lavage.^11^,^12^ Recently, this excellent bioavailability in the nodules of PC has been confirmed in human patients, and pharmacokinetic analysis showed a low drug concentration in peripheral venous blood.^13^

While these results are encouraging, it remains unclear whether PIPAC causes significant hepatic or renal toxicity. In theory, local drug delivery into the abdomen combined with the artificial intraabdominal pressure might increase the risk for first-pass hepatic toxicity and direct toxic renal parenchymal injury. Herein we report data on liver and renal toxicity from a pilot study of patients subjected to PIPAC.

## Patients and Methods

### Study Design

This is a prospective data collection (phase 0 study) within the framework of individual compassionate use of an experimental therapy as defined by article 41 nr. 2 ff AMG (German Arzneimittelgesetz). The procedures were performed at the Evangelisches Krankenhaus Bielefeld and at Marienhospital, Ruhr-University Bochum, Germany.

### Ethics

The protocol was approved by the Institutional Review Board (Ethikkommission der WW-Universität Münster and Medical Chamber of Westfalia-Lippe). The patients were extensively informed about the procedure and included in the study if they gave their written consent. The procedures were performed according to the Declaration of Helsinki and EC and German laws and regulations. In particular, occupational health safety risks were evaluated by two independent audits (data on file).

### Patients

Eight PIPAC procedures were performed in three patients at 4–6-week intervals, between November 2011 and February 2012. Patient and disease characteristics are summarized in Table 1.

**Table 1:** Patient characteristics

	Patient 1	Patient 2	Patient 3
Sex	M	M	F
Age (years)	38	45	74
Cancer localization	Gastric	Appendiceal	Ovarian
First diagnosis	2 years	2 months	9 years
Previous surgery	Gastrectomy, lymphadenectomy	Ileocecal resection, lymphadenectomy	Ovariectomy, hysterectomy, omentectomy, lymphadenectomy
Previous systemic chemotherapy	2 lines + experimental	1 line	2 lines + experimental
Reason for therapy interruption	MDR	Severe toxicity	MDR
PIPAC sessions (*n*)	2	3	3

*M* male, *F* female, *PIPAC* pressurized intraperitoneal aerosol chemotherapy, *MDR* multidrug resistance

### Therapy

After insufflation of 12 mmHg capnoperitoneum, two balloon trocars (12 mm and 5 mm; Applied Medical, Düsseldorf, Germany) were inserted into the abdominal wall. Diagnostic laparoscopy was performed, and the possibility of cytoreductive surgery excluded. Extent of peritoneal disease was documented by video recordings in all quadrants, and parietal biopsies were taken for anatomopathology, genomics studies, and functional research. Ascites was removed, and the volume documented. Then, a 10-mm nebulizer (Reger Medizintechnik, Rottweil, Germany) was connected to a high-pressure injector (Injektron 82 M; MedTron, Saarbruecken, Germany) and inserted into the abdomen. A pressurized aerosol containing cisplatin at a dose of 7.5 mg/m^2^ body surface and doxorubicin 1.5 mg/m^2^ body surface was then applied via the high-pressure injector and nebulizer. Therapeutic capnoperitoneum (TC) was maintained for 30 min at body temperature (37 °C). Then, TC was exhausted using a closed system including a particle filter into the waste air system of the hospital. Finally, trocars were retracted and laparoscopy ended. No drainage of the abdomen was applied. All surgical procedures were performed by the same surgeon (M.A.R.).

### Sampling

Peripheral venous blood was collected preoperatively and then daily until the 5th postoperative day. Blinded analysis was performed in the clinical chemistry laboratory of our hospital according to routine protocols.

### Statistical Analysis

Statistics were performed using SPSS version 14.0 software. Descriptive statistics included mean, median, percentiles, and confidence interval. Data are presented as box plots. Comparative statistics over time were performed by one-way repeated analysis of variance (ANOVA).

## Results

**Fig. 1:**
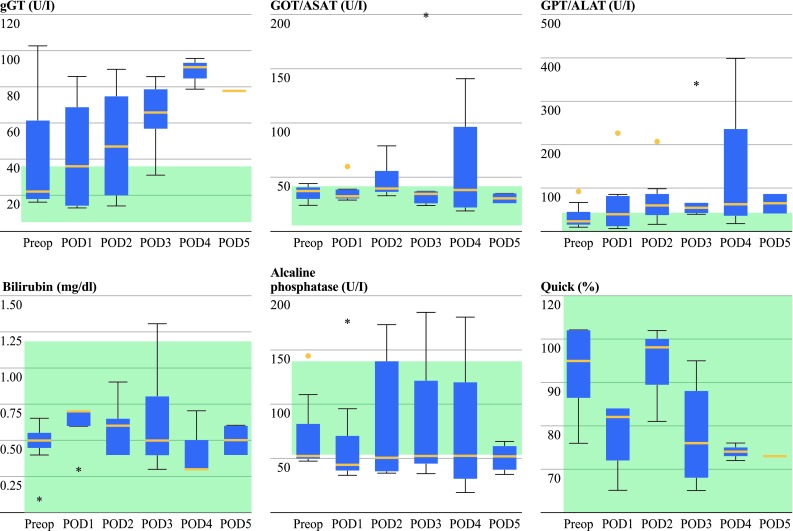
Liver toxicity: discrete liver toxicity was observed after PIPAC, with increase of serum gamma-GT levels (ANOVA, *p* < 0.05). No liver cytolysis was detected, with GOT (ASAT) and GPT (ALAT) remaining within the normal range. ANOVA: repeated analysis of variance. Normal values: gamma-GT 9–36 U/l; GOT (ASAT) 5–31 U/l; GPT (ALAT) 0–34 U/l. Liver function: Liver metabolism was not significantly impaired after PIPAC application. Liver synthesis function, as monitored by Quick-test, remained within the normal range. ANOVA: repeated analysis of variance. Normal values: alkaline phosphatase 40–150 U/l; total bilirubin <1.2 mg/dl; Quick 70–120 %. *Green shaded areas* = normal range of measured parameters

Discrete signs of liver toxicity were observed after PIPAC with cisplatin 7.5 mg/m^2^ body surface and doxorubicin 1.5 mg/m^2^ body surface (Fig. 1). First, we observed a doubling of serum gamma-GT levels with a peak on the 4th postoperative day (POD), followed by a decrease on POD 5 (one-way ANOVA, *p* = 0.22). Discrete liver cytolysis was detected, with maximal GPT (ALAT) serum level of 135 ± 177 U/l on POD 4 versus a preoperative value of 35 ± 31 U/l (*p* = 0.57). We also observed an increase of GOT (ASAT) serum levels, with a peak of 76 ± 33 U/l on POD 3 versus a preoperative value of 35 ± 8 U/l (*p* = 0.68).

Liver synthesis was also discretely impaired after PIPAC application. Quick-test dropped from 103 ± 8 % (preoperatively) to 84 ± 2 % on POD 4. However, the mean values remained within the normal range (70–100 %) (Fig. 1). Total bilirubin serum levels remained within the normal range, increasing slightly on POD 1 and then returning to the preoperative value within 4 days.

**Fig. 2:**
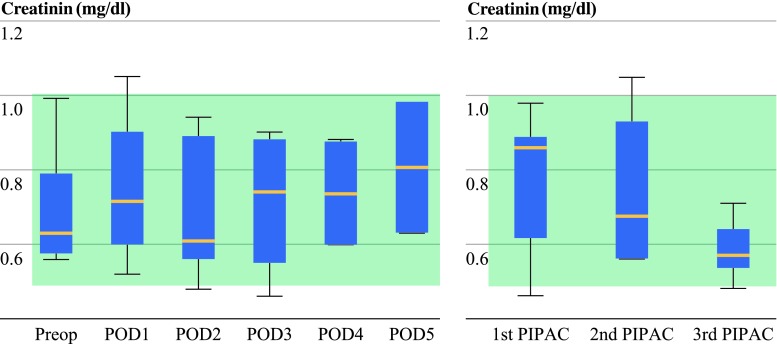
Renal function: serum creatinine levels did not increase significantly (ANOVA) after PIPAC application and remained within the normal range. ANOVA: repeated analysis of variance. Normal value: 0.5–0.9 mg/dl. Cumulative renal toxicity of repeated PIPAC with cisplatin 7.5 mg/m^2^ body surface and doxorubicin 1.5 mg/m^2^ body surface over 2–3 months of observation time. Preoperative serum creatinine value before first PIPAC (three patients), second (three patients), and third PIPAC (two patients). Creatinine levels remain within the normal range. Normal value: 0.5–0.9 mg/dl. *Green shaded areas* = normal range of measured parameters

Renal function was not impaired: Serum creatinine levels remained within the normal range (Fig. 2), with a peak of 0.75 ± 0.19 mmol/l on POD 1 versus a preoperative mean value of 0.70 ± 0.17 mmol/l.

No cumulative toxicity was observed after repeated PIPAC application at 4-week intervals. All three patients received PIPAC twice or three times. The preoperative mean serum creatinine level was not increased, as compared with the reference value before the first application, so that cumulative renal injury could be reasonably excluded (Fig. 2). A similar pattern was observed for liver toxicity: serum GOT, GPT, and bilirubin as well as Quick-test did not increase significantly with repeated NIPAC application (data not shown)

## Discussion

These pilot data obtained in the first patients treated with PIPAC worldwide show that, with the drugs above and with the dose range tested, PIPAC did not induce significant renal or hepatic toxicity. This is remarkable since application of chemotherapy was repeated twice or three times at 4-week intervals.

For hyperthermic intraperitoneal chemotherapy (HIPEC), a combination of cisplatin and doxorubicin appears to be one of the most effective available regimens with tolerable locoregional toxicity. Currently, all patients with PC managed at our institution with cytoreductive surgery (CRS) and HIPEC are given doxorubicin and cisplatin. Pharmacological aspects of intraperitoneal administration of these drugs are well known: Doxorubicin shows a much more advantageous plasma/peritoneal area under the curve (AUC) ratio than cisplatin (162 ± 113 and 20 ± 6, respectively). On the other hand, very high intraperitoneal concentrations of cisplatin can be achieved without inflicting significant systemic toxicity. Penetration of the tumor mass is greater for cisplatin than doxorubicin, as reviewed previously.^14^

After parenteral administration, cisplatin is present as an unreactive, noncharged dichloride complex in the extracellular space. This lack of electrical charge facilitates transport across the vascular wall and the cellular membrane. Within the cell, the chloride concentration is low (4 mmol/l) and the chloride ions of the complex are exchanged by OH-groups and free water molecules so that highly reactive water–hydroxide–chloride complexes are formed. These complexes have a toxic alkylating effect and cannot leave the cell anymore because of their electric charge, eventually causing cellular death. Cisplatin is mainly eliminated via the kidney, so that hepatic side-effects of cisplatin are minimal. In the kidney, the various metabolites of cisplatin reach high local concentration, and the drug has dose-dependent renal toxicity, as reviewed previously.^15^ In clinical practice and with a standard systemic cisplatin regimen (75–100 mg/m^2^ body surface), tubular function is impaired in about 30 % of patients, resulting in some cases in nonreversible tubular necrosis and chronic renal failure.^16^ We have observed in our patients comparable side-effects after HIPEC with cisplatin doses over 75 mg/m^2^ body surface (unpublished data). Parent platinum-based chemotherapeutics such as carboplatin or oxaliplatin have the same mechanism of action, but with a lower incidence of side-effects, as reviewed previously.^17^

Doxorubicin belongs to the family of anthracyclines and induces radical reactions (covalent binding to various molecules) as well as formation of superoxide radical anions (O_2_^–^) and hydrogen peroxide (H_2_O_2_). In the process of inactivation of these molecules, highly reactive and toxic hydroxide radicals (OH^–^) are generated, which in turn cause single- and double-strand DNA breaks, as reviewed previously.^15^ Cellular death after doxorubicin therapy does not occur by apoptosis, but is a sort of “dirty death” with release of toxic metabolites into the surrounding tissue, initiating a local chain reaction involving neighboring cells. This explains why extravasation of doxorubicin during intravenous delivery provokes extensive local tissue necrosis. After parenteral application, tissue uptake of doxorubicin is rapid, and elimination occurs mainly via biliary excretion after hepatic metabolism. Interestingly, doxorubicin has only moderate liver toxicity.^18^

During PIPAC, only about 10 % of a usual systemic drug dose is applied into the abdomen. Moreover, systemic drug concentration is minimal, about 1 % of a systemic dose or 5 % of a HIPEC dose.

However, the discussion should not be limited to the dose applied but should also consider particular aspects of PIPAC such as delivery in the immediate vicinity of target organs, pressure application, and hemodynamic effects, with the risk of inducing direct local toxicity.

The drug dose for our first experimental PIPAC protocol was determined based on the dosage used for intravenous delivery, with the assumption that systemic toxicity could not be more pronounced than after intravenous delivery, since systemic uptake cannot exceed 100 %. However, we were concerned about possible local side-effects within the abdomen such as bowel necrosis, and decided to use the same concentration in the chemotherapy solution as during HIPEC. Since the maximal volume that can be nebulized during PIPAC is about 200 ml, the total dose to be applied has to be limited to about 10 % of a standard HIPEC dose. In light of our first observations in human patients, it appears that this cautious protocol design was probably life-saving since the local bioavailability in PIPAC appears much higher than after HIPEC. During PIPAC, chemotherapy is nebulized into the peritoneal cavity under pressure of 12 mmHg. The rationale for increasing the intraperitoneal pressure was to create a gradient counteracting interstitial fluid pressure within tumors. Since interstitial pressure is responsible for poor penetration of chemotherapeutic drugs into tumors, bioavailability is expected to be enhanced when the intraabdominal pressure is artificially raised.^5^ This hypothesis has been confirmed in small-animal experiments, in a large-animal model, as well as ex vivo on human specimen of PC.^6^,^11^,^12^ Our results in vivo provide further proof that bioavailability in tumor nodules is excellent after PIPAC, exceeding what can be achieved with HIPEC (unpublished data).

A third topic of interest is that increasing the intraabdominal pressure impairs both portal and renal blood flow. As a consequence, renal function is decreased during capnoperitoneum, depending on the level of hydration, intraabdominal pressure, patient positioning, and procedure duration.^19^ An increase of the intraabdominal pressure by 5 mmHg (from 10 to 15 mmHg) resulted in a blood flow decrease by 39 % to the liver and by 60 % to the peritoneum. Splanchnic blood flow decreased along with operative time, in spite of constant intra-arterial pressure.^20^ On the basis of these data and our own observations, it appears reasonable to propose that PIPAC is advantageous over other delivery routes, because of limited blood inflow into the intraabdominal organs during the uptake phase. This results in limited outflow from the splanchnic circulation to the systemic compartment, which leads to high tissue bioavailability and low systemic plasma concentration. The pharmacological data collected in the first patients confirm that the systemic AUC of doxorubicin after PIPAC is only about 1 % of that of systemic administration and 5 % of that of HIPEC administration (unpublished data).

Finally, we did not observe any cumulative toxicity. PIPAC can be applied several times without any difficulties, since no therapy-related adhesions developed. This is indeed an important feature for developing effective locoregional chemotherapy regimen including several cycles and is a clear advantage over HIPEC, for which repeated application is exceptional. However, repeated administration of anthracyclines is known to induce cumulative organ toxicity; For example, application of doxorubicin is followed by severe fibrosis induction, and cardiotoxic effects are known to develop with a delay of up to 6 months, being irreversible and eventually lethal. Thus, the maximal total dose of doxorubicin applicable is limited to 550 mg/m^2^.^15^ In our first patients, no signs of cumulative renal or hepatic toxicity were observed after the second or third PIPAC administration. Moreover, no clinical symptoms of cardiac toxicity were detected, in spite of the fact that one of the patients had previously developed a life-threatening cardiac failure after systemic administration of 5-fluorouracil (5-FU).

In conclusion, the first toxicity data obtained after PIPAC are promising. No clinically relevant liver cytolysis was observed, and neither metabolic nor synthetic hepatic functions were significantly impaired. Renal function remained within the normal range. PIPAC could be repeated without inducing cumulative toxicity. Thus, it appears reasonable to propose that PIPAC causes less hepatic and renal toxicity than other chemotherapy delivery routes, due to lower therapeutic doses and favorable kinetics. While promising, the data presented here have to be considered as preliminary and need to be confirmed in future studies including appropriate dose-finding and safety studies in various cancer types and with different chemotherapeutic drugs.

## References

[1] Van Lierde S, Denys H, Peeters M (2007). Systemic chemotherapy in patients with peritoneal carcinomatosis from non colorectal origin. Cancer Treat Res..

[2] Bristow RE, Puri I, Chi DS (2009). Cytoreductive surgery for recurrent ovarian cancer: a meta-analysis. Gynecol Oncol..

[3] Zani S, Papalezova K, Stinnett S, et al. Modest advances in survival for patients with colorectal-associated peritoneal carcinomatosis in the era of modern chemotherapy. *J Surg Oncol*. 2012;18 Jul. Epub ahead of print. 10.1002/jso.23222 22811275

[4] Markman M (2003). Intraperitoneal antineoplastic drug delivery: rationale and results. Lancet Oncol.

[5] Minchinton AI, Tannock IF (2006). Drug penetration in solid tumours. Nat Rev Cancer..

[6] Dedrick RL, Flessner MF (1997). Pharmacokinetic problems in peritoneal drug administration: tissue penetration and surface exposure. J Natl Cancer Inst.

[7] Jaaback K, Johnson N, Lawrie TA. Intraperitoneal chemotherapy for the initial management of primary epithelial ovarian cancer. *Cochrane Database Syst Rev*. 2011 9 Nov;(11):CD005340. Review. 10.1002/14651858.CD005340.pub3 PMC4164826 22071822

[8] Elias D, Lefevre JH, Chevalier J (2009). Complete cytoreductive surgery plus intraperitoneal chemohyperthermia with oxaliplatin for peritoneal carcinomatosis of colorectal origin. J Clin Oncol..

[9] Macrì A, Fortugno A, Saladino E (2011). Rationale and techniques of cytoreductive surgery and peritoneal chemohyperthermia. World J Gastrointest Oncol..

[10] Reymond MA, Hu B, Garcia A (2000). Feasibility of therapeutic pneumoperitoneum in a large animal model using a microvaporisator. Surg Endosc.

[11] Solaß W, Hetzel A, Nadiradze G (2012). Intraoperative intraperitonal drug delivery using a nebulizer: rationale and pharmacokinetic results. Surg Endosc..

[12] Solass W, Herbette A, Schwarz T (2012). Therapeutic approach of human peritoneal carcinomatosis with Dbait in combination with capnoperitoneum: proof of concept. Surg Endosc..

[13] Reymond M. Therapeutic aerosolized chemotherapy for peritoneal carcinomatosis. In: Eurocancer 2012. John Libbey Eurotext, Paris 2012, 55–56.

[14] Rossi CR, Mocellin S, Pilati P (2003). Pharmacokinetics of intraperitoneal cisplatin and doxorubicin. Surg Oncol Clin North Am..

[15] Estler CE, Schmidt H (2007). Pharmakologie und Toxikologie.

[16] Dos Santos NA, Carvalho Rodrigues MA, Martins NM, et al. Cisplatin-induced nephrotoxicity and targets of nephroprotection: an update. *Arch Toxicol*. 2012;1 Mar. Epub ahead of print. 10.1007/s00204-012-0821-7 22382776

[17] Sakaeda T, Kadoyama K, Okuno Y (2011). Adverse event profiles of platinum agents: data mining of the public version of the FDA adverse event reporting system, AERS, and reproducibility of clinical observations. Int J Med Sci..

[18] Carvalho C, Santos RX, Cardoso S (2009). Doxorubicin: the good, the bad and the ugly effect. Curr Med Chem..

[19] Demyttenaere S, Feldman LS, Fried GM (2007). Effect of pneumoperitoneum on renal perfusion and function: a systematic review. Surg Endosc..

[20] Schilling MK, Redaelli C, Krahenbuhl L (1997). Splanchnic microcirculatory changes during CO_2_ laparoscopy. J Am Coll Surg.

